# {2,2-Bis[(4*S*)-4-isopropyl-4,5-dihydro-1,3-oxazol-2-yl]propane}­bis­(*N*,*N*-dimethyl­formamide)­copper(II) bis­[hexa­fluoridoanti­monate(V)]

**DOI:** 10.1107/S1600536810048658

**Published:** 2010-11-27

**Authors:** Julia Zeh, Melina Möller, Carsten Strohmann, Hans Preut, Martin Hiersemann

**Affiliations:** a Fakultät Chemie, Technische Universität Dortmund, Otto-Hahn-Strasse 6, 44221 Dortmund, Germany

## Abstract

In the title compound, [Cu(C_15_H_26_N_2_O_2_)(C_3_H_7_NO)_2_][SbF_6_]_2_, which is a potential catalyst in the catalytic asymmetric Gosteli–Claisen rearrangement, the central Cu^II^ atom is in a nearly square-planar *cis*-N_2_O_2_ environment in the cation arising from its coordination by an *N*,*N*-bidentate 2,2-bis­[(4*S*)-4-isopropyl-4,5-dihydro-1,3-oxazol-2-yl]propane ligand and two *O*-bonded *N*,*N*-dimethyl­formamide mol­ecules. Two SbF_6_^−^ anions are positioned on opposite sides of the plane through the CuN_2_O_2_ unit, generating an axially distorted CuN_2_O_2_F_2_ octa­hedral geometry for the metal ion.

## Related literature

For background to the catalytic asymmetric  Gosteli–Claisen rearrangement, see: Abraham & Hiersemann (2001 ▶); Abraham *et al.* (2001 ▶, 2004 ▶); Hiersemann & Abraham (2002 ▶). For further synthetic details, see: Evans *et al.* (1991 ▶, 1998 ▶); McKennon *et al.* (1993 ▶). For application of the catalytic asymmetric Gosteli–Claisen rearrangement, see: Körner & Hiersemann (2007 ▶); Pollex & Hiersemann (2005 ▶).
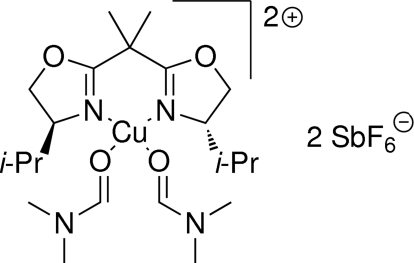


## Experimental

### 

#### Crystal data

[Cu(C_15_H_26_N_2_O_2_)(C_3_H_7_NO)_2_][SbF_6_]_2_
*M* *_r_* = 947.61
Orthorhombic, 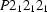

*a* = 9.7256 (2) Å
*b* = 15.2444 (3) Å
*c* = 23.2040 (5) Å
*V* = 3440.25 (12) Å^3^
*Z* = 4
Mo *K*α radiation
μ = 2.27 mm^−1^
*T* = 173 K
0.30 × 0.20 × 0.10 mm

#### Data collection

Oxford Diffraction Xcalibur S CCD diffractometer
Absorption correction: multi-scan (*CrysAlis RED*; Oxford Diffraction, 2008 ▶) *T*_min_ = 0.820, *T*_max_ = 1.000
41873 measured reflections
7479 independent reflections
6078 reflections with *I* > 2σ(*I*)
*R*_int_ = 0.046

#### Refinement

*R*[*F* ^2^ > 2σ(*F* ^2^)] = 0.027
*wR*(*F* ^2^) = 0.037
*S* = 1.04
7479 reflections
407 parameters
H-atom parameters constrained
Δρ_max_ = 0.81 e Å^−3^
Δρ_min_ = −0.52 e Å^−3^
Absolute structure: Flack (1983 ▶), 2727 Friedel pairs
Flack parameter: −0.008 (10)

### 

Data collection: *CrysAlis CCD* (Oxford Diffraction, 2008 ▶); cell refinement: *CrysAlis CCD*; data reduction: *CrysAlis CCD*; program(s) used to solve structure: *SHELXS97* (Sheldrick, 2008 ▶); program(s) used to refine structure: *SHELXL97* (Sheldrick, 2008 ▶); molecular graphics: *SHELXTL-Plus* (Sheldrick, 2008 ▶); software used to prepare material for publication: *SHELXL97* and *PLATON* (Spek, 2009 ▶).

## Supplementary Material

Crystal structure: contains datablocks I, global. DOI: 10.1107/S1600536810048658/hb5747sup1.cif

Structure factors: contains datablocks I. DOI: 10.1107/S1600536810048658/hb5747Isup2.hkl

Additional supplementary materials:  crystallographic information; 3D view; checkCIF report

## Figures and Tables

**Table 1:** Selected geometric parameters (Å, °)

Cu—O2	1.951 (2)
Cu—N4	1.962 (2)
Cu—O1	1.964 (2)
Cu—N3	1.971 (2)
Cu—F7	2.4232 (18)
Cu—F5	2.5452 (19)

**Fig. 1.**
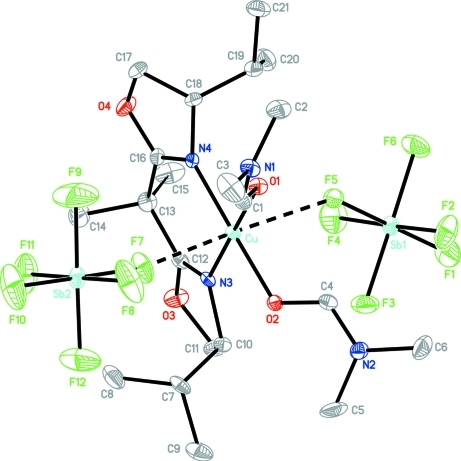
: The molecular structure of the title compound with H-atoms are omitted for clarity. Displacement ellipsoids are shown at the 30% probability level.

**Fig. 2.**
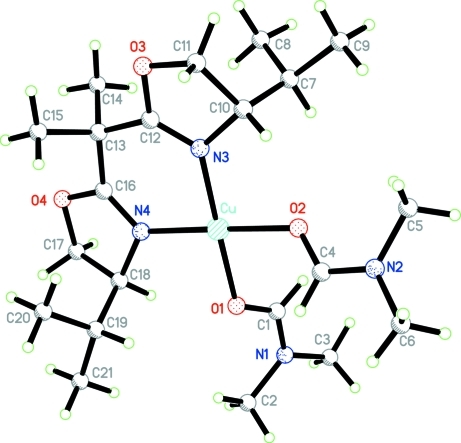
: The molecular structure of the cation [Cu(C15H26N2O2)(C3H7NO)2]2+.

## supplementary crystallographic information

### Comment

The title compound, (I), was tested as a catalyst in the catalytic asymmetric Gosteli-Claisen rearrangement (Abraham *et al.*, 2001; Abraham & Hiersemann, 2001; Hiersemann & Abraham, 2002; Abraham *et al.*, 2004). The synthesis of the title compound, (I), was accomplished according to a modified procedure of Evans *et al.* (1991, 1998). A sequence of Meyers` amino acid reduction of (S)-Valine (McKennon *et al.*, 1993), subsequent condensation with dimethyl malonic acid dichloride and *p*-TsCl catalyzed cyclization provided the (*S,S*)-^i^Pr-box ligand. Treatment of the box ligand with CuCl_2_ and subsequent anion metathesis with AgSbF_6_ provided [Cu{(*S,S*)-^i^Pr-box}](SbF_6_)_2_ (Evans *et al.*, 1998). Addition of 2 eq of DMF to a solution of [Cu{(*S,S*)-^i^Pr-box}](SbF_6_)_2_ in 1,2-dichloroethane afforded [Cu{(*S,S*)-^i^Pr-box}(dmf)_2_](SbF_6_)_2_. Crystallization was achieved by vapor diffusion recrystallization at 243 K.

### Experimental

To a solution of [Cu{(*S,S*)-^i^Pr-box}](SbF_6_)_2_ (78.1 mg, 0.094 mmol, 1 eq) in dry 1,2-dichloroethane (1 ml) under argon atmosphere was added DMF (14.5 µ*L*, 0.188 mmol, 2 eq) by a microliter syringe and the resulting deep blue solution was stirred for 15 min at room temperature. Subsequent cooling to 243 K provided (I) as deep blue blocks.

### Crystal data

**Table:**

[Cu(C_15_H_26_N_2_O_2_)(C_3_H_7_NO)_2_][SbF_6_]_2_	*F*(000) = 1860
*M**_r_* = 947.61	*D*_x_ = 1.830 Mg m^−^^3^
Orthorhombic, *P*2_1_2_1_2_1_	Mo *K*α radiation, λ = 0.71073 Å
Hall symbol: P 2ac 2ab	Cell parameters from 18634 reflections
*a* = 9.7256 (2) Å	θ = 2.2–29.1°
*b* = 15.2444 (3) Å	µ = 2.27 mm^−^^1^
*c* = 23.2040 (5) Å	*T* = 173 K
*V* = 3440.25 (12) Å^3^	Block, blue
*Z* = 4	0.30 × 0.20 × 0.10 mm

### Data collection

**Table:**

Oxford Diffraction Xcalibur S CCD diffractometer	7479 independent reflections
Radiation source: Enhance (Mo) X-ray Source	6078 reflections with *I* > 2σ(*I*)
graphite	*R*_int_ = 0.046
Detector resolution: 16.0560 pixels mm^-1^	θ_max_ = 27.0°, θ_min_ = 2.2°
ω scans	*h* = −12→13
Absorption correction: multi-scan (*CrysAlis RED*; Oxford Diffraction, 2008)	*k* = −20→20
*T*_min_ = 0.820, *T*_max_ = 1.000	*l* = −29→29
41873 measured reflections	

### Refinement

**Table:**

Refinement on *F*^2^	Secondary atom site location: difference Fourier map
Least-squares matrix: full	Hydrogen site location: inferred from neighbouring sites
*R*[*F*^2^ > 2σ(*F*^2^)] = 0.027	H-atom parameters constrained
*wR*(*F*^2^) = 0.037	*w* = 1/[σ^2^(*F*_o_^2^) + (0.0104*P*)^2^] where *P* = (*F*_o_^2^ + 2*F*_c_^2^)/3
*S* = 1.04	(Δ/σ)_max_ = 0.002
7479 reflections	Δρ_max_ = 0.81 e Å^−^^3^
407 parameters	Δρ_min_ = −0.52 e Å^−^^3^
0 restraints	Absolute structure: Flack (1983), 3264 Friedel pairs
Primary atom site location: structure-invariant direct methods	Flack parameter: −0.008 (10)

### Special details

**Table:**

Experimental. CrysAlis RED, Oxford Diffraction Ltd., Version 1.171.32.37 (release 24-10-2008) Empirical absorption correction using sperical harmonics, implemented in SCALE3 ABSPACK scaling algorithm.
Geometry. All e.s.d.'s (except the e.s.d. in the dihedral angle between two l.s. planes) are estimated using the full covariance matrix. The cell e.s.d.'s are taken into account individually in the estimation of e.s.d.'s in distances, angles and torsion angles; correlations between e.s.d.'s in cell parameters are only used when they are defined by crystal symmetry. An approximate (isotropic) treatment of cell e.s.d.'s is used for estimating e.s.d.'s involving l.s. planes.
Refinement. Refinement of *F*^2^ against ALL reflections. The weighted *R*-factor *wR* and goodness of fit *S* are based on *F*^2^, conventional *R*-factors *R* are based on *F*, with *F* set to zero for negative *F*^2^. The threshold expression of *F*^2^ > σ(*F*^2^) is used only for calculating *R*-factors(gt) *etc*. and is not relevant to the choice of reflections for refinement. *R*-factors based on *F*^2^ are statistically about twice as large as those based on *F*, and *R*- factors based on ALL data will be even larger.

### Fractional atomic coordinates and isotropic or equivalent isotropic displacement parameters (Å^2^)

**Table:**

	*x*	*y*	*z*	*U*_iso_*/*U*_eq_	
C1	0.5137 (4)	−0.0127 (2)	0.49613 (13)	0.0289 (9)	
H1	0.4400	−0.0272	0.5213	0.035*	
C2	0.6042 (3)	−0.0081 (2)	0.40077 (13)	0.0401 (10)	
H2A	0.6808	0.0202	0.4210	0.060*	
H2B	0.5682	0.0317	0.3712	0.060*	
H2C	0.6367	−0.0622	0.3825	0.060*	
C3	0.3714 (4)	−0.0676 (3)	0.41833 (15)	0.0533 (12)	
H3A	0.3091	−0.0825	0.4500	0.080*	
H3B	0.3947	−0.1209	0.3968	0.080*	
H3C	0.3264	−0.0256	0.3925	0.080*	
C4	0.4588 (3)	0.1619 (2)	0.58081 (13)	0.0256 (8)	
H4	0.5160	0.1732	0.5484	0.031*	
C5	0.2758 (4)	0.2059 (3)	0.64305 (15)	0.0542 (12)	
H5A	0.2778	0.1441	0.6548	0.081*	
H5B	0.1810	0.2231	0.6343	0.081*	
H5C	0.3114	0.2425	0.6744	0.081*	
C6	0.3425 (4)	0.2977 (2)	0.55892 (15)	0.0454 (10)	
H6A	0.3591	0.3486	0.5837	0.068*	
H6B	0.2484	0.3001	0.5439	0.068*	
H6C	0.4078	0.2982	0.5268	0.068*	
C7	0.4543 (3)	0.0165 (2)	0.74066 (13)	0.0287 (9)	
H7	0.4068	0.0057	0.7031	0.034*	
C8	0.4871 (4)	−0.0730 (2)	0.76668 (15)	0.0483 (12)	
H8A	0.5467	−0.1059	0.7403	0.073*	
H8B	0.4015	−0.1057	0.7728	0.073*	
H8C	0.5342	−0.0651	0.8036	0.073*	
C9	0.3533 (4)	0.0675 (3)	0.77929 (14)	0.0444 (11)	
H9A	0.3973	0.0803	0.8164	0.067*	
H9B	0.2707	0.0320	0.7857	0.067*	
H9C	0.3278	0.1226	0.7604	0.067*	
C10	0.5804 (3)	0.0722 (2)	0.72801 (12)	0.0211 (8)	
H10	0.5518	0.1305	0.7123	0.025*	
C11	0.6784 (4)	0.0848 (2)	0.77903 (13)	0.0324 (9)	
H11A	0.7194	0.1443	0.7786	0.039*	
H11B	0.6298	0.0761	0.8161	0.039*	
C12	0.7743 (3)	−0.0033 (2)	0.71476 (13)	0.0253 (8)	
C13	0.8862 (3)	−0.0654 (2)	0.69651 (13)	0.0299 (9)	
C14	0.8660 (4)	−0.1533 (2)	0.72814 (14)	0.0482 (11)	
H14A	0.8620	−0.1428	0.7698	0.072*	
H14B	0.9432	−0.1924	0.7194	0.072*	
H14C	0.7800	−0.1806	0.7153	0.072*	
C15	1.0259 (3)	−0.0252 (3)	0.71247 (15)	0.0457 (11)	
H15A	1.0368	0.0312	0.6927	0.069*	
H15B	1.0997	−0.0650	0.7006	0.069*	
H15C	1.0303	−0.0161	0.7542	0.069*	
C16	0.8874 (3)	−0.0824 (2)	0.63266 (13)	0.0241 (8)	
C17	0.9624 (4)	−0.1531 (2)	0.55485 (14)	0.0317 (9)	
H17A	0.9144	−0.2091	0.5469	0.038*	
H17B	1.0517	−0.1530	0.5343	0.038*	
C18	0.8747 (3)	−0.0751 (2)	0.53675 (13)	0.0221 (8)	
H18	0.7999	−0.0951	0.5103	0.027*	
C19	0.9522 (4)	0.0016 (2)	0.50941 (13)	0.0295 (9)	
H19	0.8849	0.0508	0.5049	0.035*	
C20	1.0693 (4)	0.0358 (2)	0.54629 (15)	0.0429 (10)	
H20A	1.0330	0.0564	0.5833	0.064*	
H20B	1.1149	0.0845	0.5264	0.064*	
H20C	1.1358	−0.0114	0.5530	0.064*	
C21	1.0018 (4)	−0.0230 (3)	0.44894 (15)	0.0426 (10)	
H21A	1.0490	0.0273	0.4316	0.064*	
H21B	0.9227	−0.0393	0.4251	0.064*	
H21C	1.0654	−0.0726	0.4515	0.064*	
Cu	0.64767 (4)	0.02331 (2)	0.602048 (15)	0.01965 (9)	
F1	0.8666 (3)	0.37550 (14)	0.65651 (10)	0.0660 (8)	
F2	0.7212 (2)	0.32927 (15)	0.56382 (10)	0.0693 (8)	
F3	0.6661 (2)	0.25671 (14)	0.66418 (9)	0.0605 (7)	
F4	0.9252 (2)	0.20713 (17)	0.67247 (9)	0.0705 (7)	
F5	0.77901 (19)	0.16435 (12)	0.58166 (8)	0.0398 (5)	
F6	0.97787 (19)	0.28174 (17)	0.57245 (9)	0.0569 (6)	
F7	0.5062 (2)	−0.10547 (12)	0.61729 (9)	0.0539 (6)	
F8	0.3044 (3)	−0.15540 (19)	0.54937 (11)	0.0903 (10)	
F9	0.5315 (3)	−0.2408 (2)	0.55262 (12)	0.1229 (13)	
F10	0.3038 (3)	−0.31014 (16)	0.59552 (11)	0.1034 (10)	
F11	0.5076 (3)	−0.26059 (18)	0.66505 (11)	0.0897 (9)	
F12	0.2818 (2)	−0.1733 (2)	0.66203 (12)	0.0942 (10)	
N1	0.4964 (3)	−0.02873 (19)	0.44157 (11)	0.0281 (7)	
N2	0.3604 (3)	0.21768 (18)	0.59222 (10)	0.0273 (7)	
N3	0.6737 (3)	0.02687 (16)	0.68626 (9)	0.0188 (6)	
N4	0.8145 (3)	−0.04765 (16)	0.59293 (10)	0.0192 (6)	
O1	0.6184 (2)	0.01985 (15)	0.51831 (8)	0.0240 (5)	
O2	0.4822 (2)	0.09465 (13)	0.60992 (9)	0.0232 (5)	
O3	0.7822 (2)	0.01878 (17)	0.77070 (9)	0.0345 (6)	
O4	0.9819 (2)	−0.14044 (14)	0.61644 (10)	0.0347 (6)	
Sb1	0.82253 (2)	0.270184 (15)	0.619240 (9)	0.02832 (6)	
Sb2	0.40579 (2)	−0.208325 (15)	0.607463 (10)	0.03231 (6)	

### Atomic displacement parameters (Å^2^)

**Table:**

	*U*^11^	*U*^22^	*U*^33^	*U*^12^	*U*^13^	*U*^23^
C1	0.037 (2)	0.032 (2)	0.0181 (19)	0.007 (2)	0.0039 (16)	0.0065 (17)
C2	0.046 (2)	0.056 (3)	0.0185 (18)	0.013 (2)	0.0007 (17)	−0.0003 (17)
C3	0.058 (3)	0.071 (3)	0.030 (2)	−0.024 (3)	−0.0164 (19)	0.002 (2)
C4	0.026 (2)	0.032 (2)	0.0195 (18)	−0.0009 (19)	0.0029 (15)	−0.0005 (17)
C5	0.046 (2)	0.073 (3)	0.043 (2)	0.040 (3)	0.0168 (18)	0.009 (2)
C6	0.051 (3)	0.034 (2)	0.051 (2)	0.017 (2)	−0.004 (2)	0.0040 (19)
C7	0.033 (2)	0.041 (2)	0.0115 (17)	−0.015 (2)	0.0025 (14)	0.0001 (16)
C8	0.065 (3)	0.048 (3)	0.032 (2)	−0.015 (2)	0.011 (2)	0.007 (2)
C9	0.046 (3)	0.061 (3)	0.026 (2)	−0.014 (2)	0.0115 (18)	−0.0117 (19)
C10	0.0221 (19)	0.028 (2)	0.0135 (16)	0.0001 (18)	−0.0008 (14)	−0.0016 (14)
C11	0.034 (2)	0.041 (2)	0.0223 (18)	0.004 (2)	0.0015 (17)	−0.0109 (16)
C12	0.025 (2)	0.032 (2)	0.0185 (18)	−0.0036 (18)	−0.0003 (14)	0.0049 (16)
C13	0.032 (2)	0.035 (2)	0.0219 (18)	0.005 (2)	−0.0057 (15)	0.0043 (16)
C14	0.066 (3)	0.047 (3)	0.032 (2)	0.014 (2)	0.000 (2)	0.0125 (19)
C15	0.039 (2)	0.072 (3)	0.027 (2)	0.015 (2)	−0.0120 (17)	−0.013 (2)
C16	0.025 (2)	0.0183 (18)	0.0286 (19)	−0.0002 (17)	0.0016 (15)	0.0023 (15)
C17	0.038 (2)	0.023 (2)	0.034 (2)	0.0064 (19)	0.0071 (17)	−0.0083 (17)
C18	0.020 (2)	0.023 (2)	0.0231 (18)	0.0025 (17)	0.0029 (13)	−0.0055 (15)
C19	0.034 (2)	0.030 (2)	0.0244 (19)	0.0044 (19)	0.0065 (15)	−0.0016 (16)
C20	0.046 (3)	0.038 (2)	0.044 (2)	−0.010 (2)	0.008 (2)	0.000 (2)
C21	0.042 (2)	0.050 (3)	0.036 (2)	0.000 (2)	0.0171 (18)	−0.004 (2)
Cu	0.0244 (2)	0.0223 (2)	0.01230 (19)	0.00190 (19)	0.00036 (16)	0.00085 (17)
F1	0.089 (2)	0.0442 (14)	0.0649 (17)	−0.0308 (14)	0.0121 (13)	−0.0198 (12)
F2	0.0860 (19)	0.0492 (16)	0.0727 (18)	0.0242 (14)	−0.0256 (14)	0.0122 (13)
F3	0.0603 (13)	0.0506 (15)	0.0704 (15)	−0.0135 (13)	0.0353 (12)	−0.0259 (12)
F4	0.0802 (17)	0.0779 (18)	0.0534 (14)	0.0076 (17)	−0.0219 (12)	0.0184 (15)
F5	0.0443 (13)	0.0282 (12)	0.0470 (13)	−0.0084 (10)	0.0141 (9)	−0.0097 (10)
F6	0.0401 (12)	0.0687 (18)	0.0618 (14)	−0.0192 (14)	0.0148 (10)	−0.0022 (14)
F7	0.0751 (15)	0.0443 (13)	0.0422 (14)	−0.0327 (11)	−0.0080 (12)	0.0008 (12)
F8	0.0765 (19)	0.108 (2)	0.0861 (19)	−0.0432 (18)	−0.0446 (16)	0.0576 (17)
F9	0.123 (2)	0.145 (3)	0.101 (2)	−0.017 (2)	0.0507 (19)	−0.077 (2)
F10	0.166 (3)	0.0594 (18)	0.085 (2)	−0.0660 (19)	−0.0287 (19)	0.0046 (15)
F11	0.097 (2)	0.068 (2)	0.105 (2)	0.0046 (16)	−0.0373 (16)	0.0335 (18)
F12	0.0541 (17)	0.135 (3)	0.094 (2)	−0.0123 (17)	0.0283 (14)	−0.028 (2)
N1	0.0313 (17)	0.0369 (19)	0.0161 (15)	0.0008 (16)	−0.0018 (13)	0.0025 (14)
N2	0.0270 (15)	0.0310 (17)	0.0238 (14)	0.0080 (16)	−0.0014 (11)	0.0036 (13)
N3	0.0200 (15)	0.0206 (15)	0.0159 (13)	−0.0055 (15)	0.0026 (12)	0.0018 (11)
N4	0.0227 (15)	0.0151 (14)	0.0197 (14)	0.0004 (14)	0.0028 (12)	0.0004 (11)
O1	0.0245 (14)	0.0312 (14)	0.0164 (12)	0.0012 (12)	−0.0031 (9)	−0.0015 (10)
O2	0.0267 (12)	0.0247 (12)	0.0184 (12)	0.0055 (10)	0.0031 (10)	0.0070 (11)
O3	0.0385 (15)	0.0518 (18)	0.0131 (12)	0.0099 (14)	−0.0065 (10)	−0.0055 (12)
O4	0.0393 (14)	0.0307 (14)	0.0340 (15)	0.0170 (12)	−0.0026 (12)	−0.0011 (13)
Sb1	0.03023 (13)	0.02309 (12)	0.03164 (13)	−0.00408 (12)	−0.00031 (11)	−0.00031 (11)
Sb2	0.04411 (15)	0.02205 (13)	0.03076 (13)	−0.00479 (12)	−0.00402 (11)	−0.00098 (11)

### Geometric parameters (Å, °)

**Table:**

C1—O1	1.245 (4)	C13—C14	1.540 (5)
C1—N1	1.300 (4)	C14—H14A	0.9800
C1—H1	0.9500	C14—H14B	0.9800
C2—N1	1.448 (4)	C14—H14C	0.9800
C2—H2A	0.9800	C15—H15A	0.9800
C2—H2B	0.9800	C15—H15B	0.9800
C2—H2C	0.9800	C15—H15C	0.9800
C3—N1	1.456 (4)	C16—N4	1.278 (4)
C3—H3A	0.9800	C16—O4	1.330 (4)
C3—H3B	0.9800	C17—O4	1.455 (4)
C3—H3C	0.9800	C17—C18	1.523 (4)
C4—O2	1.249 (4)	C17—H17A	0.9900
C4—N2	1.307 (4)	C17—H17B	0.9900
C4—H4	0.9500	C18—N4	1.489 (4)
C5—N2	1.450 (4)	C18—C19	1.529 (4)
C5—H5A	0.9800	C18—H18	1.0000
C5—H5B	0.9800	C19—C20	1.517 (4)
C5—H5C	0.9800	C19—C21	1.530 (4)
C6—N2	1.454 (4)	C19—H19	1.0000
C6—H6A	0.9800	C20—H20A	0.9800
C6—H6B	0.9800	C20—H20B	0.9800
C6—H6C	0.9800	C20—H20C	0.9800
C7—C10	1.521 (4)	C21—H21A	0.9800
C7—C8	1.526 (5)	C21—H21B	0.9800
C7—C9	1.540 (5)	C21—H21C	0.9800
C7—H7	1.0000	Cu—O2	1.951 (2)
C8—H8A	0.9800	Cu—N4	1.962 (2)
C8—H8B	0.9800	Cu—O1	1.964 (2)
C8—H8C	0.9800	Cu—N3	1.971 (2)
C9—H9A	0.9800	Cu—F7	2.4232 (18)
C9—H9B	0.9800	Cu—F5	2.5452 (19)
C9—H9C	0.9800	F1—Sb1	1.873 (2)
C10—N3	1.497 (4)	F2—Sb1	1.854 (2)
C10—C11	1.532 (4)	F3—Sb1	1.8560 (18)
C10—H10	1.0000	F4—Sb1	1.856 (2)
C11—O3	1.439 (4)	F5—Sb1	1.8822 (18)
C11—H11A	0.9900	F6—Sb1	1.8687 (19)
C11—H11B	0.9900	F7—Sb2	1.8613 (18)
C12—N3	1.268 (4)	F8—Sb2	1.855 (2)
C12—O3	1.343 (4)	F9—Sb2	1.832 (2)
C12—C13	1.503 (5)	F10—Sb2	1.863 (2)
C13—C16	1.504 (4)	F11—Sb2	1.844 (2)
C13—C15	1.536 (4)	F12—Sb2	1.828 (2)
			
O1—C1—N1	125.7 (3)	H17A—C17—H17B	109.0
O1—C1—H1	117.1	N4—C18—C17	101.4 (2)
N1—C1—H1	117.1	N4—C18—C19	110.0 (3)
N1—C2—H2A	109.5	C17—C18—C19	115.8 (3)
N1—C2—H2B	109.5	N4—C18—H18	109.7
H2A—C2—H2B	109.5	C17—C18—H18	109.7
N1—C2—H2C	109.5	C19—C18—H18	109.7
H2A—C2—H2C	109.5	C20—C19—C18	113.5 (3)
H2B—C2—H2C	109.5	C20—C19—C21	111.4 (3)
N1—C3—H3A	109.5	C18—C19—C21	110.4 (3)
N1—C3—H3B	109.5	C20—C19—H19	107.1
H3A—C3—H3B	109.5	C18—C19—H19	107.1
N1—C3—H3C	109.5	C21—C19—H19	107.1
H3A—C3—H3C	109.5	C19—C20—H20A	109.5
H3B—C3—H3C	109.5	C19—C20—H20B	109.5
O2—C4—N2	123.9 (3)	H20A—C20—H20B	109.5
O2—C4—H4	118.1	C19—C20—H20C	109.5
N2—C4—H4	118.1	H20A—C20—H20C	109.5
N2—C5—H5A	109.5	H20B—C20—H20C	109.5
N2—C5—H5B	109.5	C19—C21—H21A	109.5
H5A—C5—H5B	109.5	C19—C21—H21B	109.5
N2—C5—H5C	109.5	H21A—C21—H21B	109.5
H5A—C5—H5C	109.5	C19—C21—H21C	109.5
H5B—C5—H5C	109.5	H21A—C21—H21C	109.5
N2—C6—H6A	109.5	H21B—C21—H21C	109.5
N2—C6—H6B	109.5	O2—Cu—N4	179.10 (10)
H6A—C6—H6B	109.5	O2—Cu—O1	89.32 (9)
N2—C6—H6C	109.5	N4—Cu—O1	89.90 (9)
H6A—C6—H6C	109.5	O2—Cu—N3	89.86 (10)
H6B—C6—H6C	109.5	N4—Cu—N3	90.92 (10)
C10—C7—C8	114.1 (3)	O1—Cu—N3	179.05 (10)
C10—C7—C9	110.1 (3)	O2—Cu—F7	88.26 (8)
C8—C7—C9	110.8 (3)	N4—Cu—F7	92.21 (9)
C10—C7—H7	107.2	O1—Cu—F7	92.32 (8)
C8—C7—H7	107.2	N3—Cu—F7	87.16 (9)
C9—C7—H7	107.2	O2—Cu—F5	87.74 (8)
C7—C8—H8A	109.5	N4—Cu—F5	91.75 (8)
C7—C8—H8B	109.5	O1—Cu—F5	84.92 (8)
H8A—C8—H8B	109.5	N3—Cu—F5	95.54 (8)
C7—C8—H8C	109.5	F7—Cu—F5	175.16 (7)
H8A—C8—H8C	109.5	Sb1—F5—Cu	138.67 (9)
H8B—C8—H8C	109.5	Sb2—F7—Cu	164.27 (12)
C7—C9—H9A	109.5	C1—N1—C2	120.1 (3)
C7—C9—H9B	109.5	C1—N1—C3	123.0 (3)
H9A—C9—H9B	109.5	C2—N1—C3	116.8 (3)
C7—C9—H9C	109.5	C4—N2—C5	120.0 (3)
H9A—C9—H9C	109.5	C4—N2—C6	121.7 (3)
H9B—C9—H9C	109.5	C5—N2—C6	117.9 (3)
N3—C10—C7	110.8 (3)	C12—N3—C10	107.3 (2)
N3—C10—C11	100.4 (2)	C12—N3—Cu	127.3 (2)
C7—C10—C11	115.0 (3)	C10—N3—Cu	125.2 (2)
N3—C10—H10	110.1	C16—N4—C18	107.3 (3)
C7—C10—H10	110.1	C16—N4—Cu	127.6 (2)
C11—C10—H10	110.1	C18—N4—Cu	125.06 (19)
O3—C11—C10	104.2 (2)	C1—O1—Cu	122.6 (2)
O3—C11—H11A	110.9	C4—O2—Cu	123.9 (2)
C10—C11—H11A	110.9	C12—O3—C11	105.4 (2)
O3—C11—H11B	110.9	C16—O4—C17	106.0 (2)
C10—C11—H11B	110.9	F2—Sb1—F3	90.45 (11)
H11A—C11—H11B	108.9	F2—Sb1—F4	177.53 (11)
N3—C12—O3	117.2 (3)	F3—Sb1—F4	90.55 (10)
N3—C12—C13	129.8 (3)	F2—Sb1—F6	88.91 (11)
O3—C12—C13	112.9 (3)	F3—Sb1—F6	178.42 (10)
C12—C13—C16	113.1 (3)	F4—Sb1—F6	90.03 (10)
C12—C13—C15	108.7 (3)	F2—Sb1—F1	91.46 (11)
C16—C13—C15	107.4 (3)	F3—Sb1—F1	91.31 (9)
C12—C13—C14	108.8 (3)	F4—Sb1—F1	90.78 (11)
C16—C13—C14	108.7 (3)	F6—Sb1—F1	90.14 (10)
C15—C13—C14	110.2 (3)	F2—Sb1—F5	88.61 (10)
C13—C14—H14A	109.5	F3—Sb1—F5	88.92 (8)
C13—C14—H14B	109.5	F4—Sb1—F5	89.16 (10)
H14A—C14—H14B	109.5	F6—Sb1—F5	89.63 (9)
C13—C14—H14C	109.5	F1—Sb1—F5	179.76 (10)
H14A—C14—H14C	109.5	F12—Sb2—F9	178.67 (15)
H14B—C14—H14C	109.5	F12—Sb2—F11	88.77 (13)
C13—C15—H15A	109.5	F9—Sb2—F11	91.63 (14)
C13—C15—H15B	109.5	F12—Sb2—F8	91.47 (13)
H15A—C15—H15B	109.5	F9—Sb2—F8	88.12 (14)
C13—C15—H15C	109.5	F11—Sb2—F8	179.62 (12)
H15A—C15—H15C	109.5	F12—Sb2—F7	90.88 (11)
H15B—C15—H15C	109.5	F9—Sb2—F7	87.85 (12)
N4—C16—O4	117.1 (3)	F11—Sb2—F7	89.62 (11)
N4—C16—C13	129.4 (3)	F8—Sb2—F7	90.09 (10)
O4—C16—C13	113.5 (3)	F12—Sb2—F10	89.71 (13)
O4—C17—C18	103.9 (2)	F9—Sb2—F10	91.55 (14)
O4—C17—H17A	111.0	F11—Sb2—F10	91.94 (12)
C18—C17—H17A	111.0	F8—Sb2—F10	88.36 (12)
O4—C17—H17B	111.0	F7—Sb2—F10	178.35 (11)
C18—C17—H17B	111.0		
			
C8—C7—C10—N3	59.8 (3)	F7—Cu—N3—C12	97.1 (3)
C9—C7—C10—N3	−174.9 (3)	F5—Cu—N3—C12	−86.9 (3)
C8—C7—C10—C11	−53.1 (4)	O2—Cu—N3—C10	0.8 (2)
C9—C7—C10—C11	72.1 (4)	N4—Cu—N3—C10	−179.7 (2)
N3—C10—C11—O3	−22.7 (3)	F7—Cu—N3—C10	−87.5 (2)
C7—C10—C11—O3	96.3 (3)	F5—Cu—N3—C10	88.5 (2)
N3—C12—C13—C16	9.9 (5)	O4—C16—N4—C18	−8.3 (4)
O3—C12—C13—C16	−173.6 (3)	C13—C16—N4—C18	169.4 (3)
N3—C12—C13—C15	129.1 (4)	O4—C16—N4—Cu	168.4 (2)
O3—C12—C13—C15	−54.4 (4)	C13—C16—N4—Cu	−13.8 (5)
N3—C12—C13—C14	−110.9 (4)	C17—C18—N4—C16	17.6 (3)
O3—C12—C13—C14	65.6 (4)	C19—C18—N4—C16	−105.6 (3)
C12—C13—C16—N4	5.2 (5)	C17—C18—N4—Cu	−159.3 (2)
C15—C13—C16—N4	−114.8 (4)	C19—C18—N4—Cu	77.5 (3)
C14—C13—C16—N4	126.0 (4)	O1—Cu—N4—C16	−171.4 (3)
C12—C13—C16—O4	−177.0 (3)	N3—Cu—N4—C16	8.1 (3)
C15—C13—C16—O4	63.0 (4)	F7—Cu—N4—C16	−79.1 (3)
C14—C13—C16—O4	−56.2 (4)	F5—Cu—N4—C16	103.7 (3)
O4—C17—C18—N4	−20.0 (3)	O1—Cu—N4—C18	4.8 (2)
O4—C17—C18—C19	99.0 (3)	N3—Cu—N4—C18	−175.7 (2)
N4—C18—C19—C20	59.9 (4)	F7—Cu—N4—C18	97.1 (2)
C17—C18—C19—C20	−54.3 (4)	F5—Cu—N4—C18	−80.1 (2)
N4—C18—C19—C21	−174.2 (3)	N1—C1—O1—Cu	−169.4 (3)
C17—C18—C19—C21	71.6 (4)	O2—Cu—O1—C1	−61.1 (3)
O2—Cu—F5—Sb1	62.36 (16)	N4—Cu—O1—C1	119.4 (3)
N4—Cu—F5—Sb1	−118.37 (16)	F7—Cu—O1—C1	27.2 (3)
O1—Cu—F5—Sb1	151.89 (16)	F5—Cu—O1—C1	−148.9 (3)
N3—Cu—F5—Sb1	−27.28 (17)	N2—C4—O2—Cu	−168.2 (2)
O2—Cu—F7—Sb2	106.6 (4)	O1—Cu—O2—C4	−50.1 (2)
N4—Cu—F7—Sb2	−72.7 (4)	N3—Cu—O2—C4	130.4 (3)
O1—Cu—F7—Sb2	17.3 (4)	F7—Cu—O2—C4	−142.4 (3)
N3—Cu—F7—Sb2	−163.5 (4)	F5—Cu—O2—C4	34.9 (2)
O1—C1—N1—C2	−0.3 (5)	N3—C12—O3—C11	−9.1 (4)
O1—C1—N1—C3	179.5 (3)	C13—C12—O3—C11	173.9 (3)
O2—C4—N2—C5	3.6 (5)	C10—C11—O3—C12	20.0 (3)
O2—C4—N2—C6	176.4 (3)	N4—C16—O4—C17	−5.6 (4)
O3—C12—N3—C10	−6.7 (4)	C13—C16—O4—C17	176.3 (3)
C13—C12—N3—C10	169.7 (3)	C18—C17—O4—C16	16.4 (3)
O3—C12—N3—Cu	169.3 (2)	Cu—F5—Sb1—F2	−116.14 (16)
C13—C12—N3—Cu	−14.2 (5)	Cu—F5—Sb1—F3	−25.67 (15)
C7—C10—N3—C12	−103.8 (3)	Cu—F5—Sb1—F4	64.90 (15)
C11—C10—N3—C12	18.2 (3)	Cu—F5—Sb1—F6	154.94 (15)
C7—C10—N3—Cu	80.0 (3)	Cu—F7—Sb2—F12	−145.8 (4)
C11—C10—N3—Cu	−158.0 (2)	Cu—F7—Sb2—F9	33.8 (4)
O2—Cu—N3—C12	−174.6 (3)	Cu—F7—Sb2—F11	125.5 (4)
N4—Cu—N3—C12	4.9 (3)	Cu—F7—Sb2—F8	−54.3 (4)
